# DeBo: Contrast enhancement for image registration using binary differential evolution and bat optimization

**DOI:** 10.1371/journal.pone.0315902

**Published:** 2024-12-26

**Authors:** Muhammad Adeel Akram, Tallha Akram, Umer Javed, Muhammad Rafiq, Mehvish Naz, Di He

**Affiliations:** 1 Department of Electrical and Computer Engineering, COMSATS University Islamabad, Wah Campus, Wah, Pakistan; 2 Department of Information Systems, College of Computer Engineering and Sciences, Prince Sattam bin Abdulaziz University, Alkharj, Saudi Arabia; 3 Department of Mathematics, COMSATS University Islamabad, Wah Campus, Wah, Pakistan; 4 Shanghai Key Laboratory of Navigation and Location-based Services, School of Sensing Science and Engineering, School of Electronic Information and Electrical Engineering, Shanghai Jiao Tong University, Shanghai, P.R. China; Mirpur University of Science and Technology, PAKISTAN

## Abstract

Image registration has demonstrated its significance as an essential tool for target recognition, classification, tracking, and damage assessment during natural catastrophes. The image registration process relies on the identification of numerous reliable features; thus, low resolutions, poor lighting conditions, and low image contrast substantially diminish the number of dependable features available for registration. Contrast stretching enhances image quality, facilitating the object detection process. In this study, we proposed a hybrid binary differential evolution and BAT optimization model to enhance contrast stretching by optimizing a decision variables in the transformation function. To validate its efficiency, the proposed approach is utilized as a preprocessor before feature extraction in image registration. Cross-comparison of detected features of enhanced images verses the original images during image registration validate the improvements in the image registration process.

## Introduction

Image registration involves the alignment of multiple images (two or more) that are taken from the same scene under different conditions, for instance, from different sensors, from different viewpoints, under different illumination conditions, or at different times, etc. The alignment process is the fundamental requirement in diverse applications, including image stitching, environmental monitoring [1], change detection [2], precision agriculture [3, 4], map updating [5] and number of others.

Image registration is broadly categorized into intensity-based and feature-based [6]. In the intensity-based registration process, intensity is used to relate the reference and target images, such as mutual information [7] and correlation coefficient [8, 9]. The selection of the transformation model and the similarity function are the critical choices that affects the performance of the intensity-based approaches. However, a large number of resources are required to handle a substantial amount of transformation parameters. Moreover, the similarity function involves calculation between pixels of each image, which is not a computationally economical task. Furthermore, intensity-based image registration algorithms are more susceptible to noise, scale variations, illumination changes and contrast [6, 10]. Furthermore, intensity-based techniques cannot be used for multi-spectral image registration because the intensity changes non-linearly in multi-spectral images [11].

Feature-based techniques perform image registration by matching the distinguishable features extracted from both images. Corners are considered the most reliable features in feature-based image registration [1] since they are invariant to image geometry and can be observed by the human eye [12]. Therefore, feature-based image registration methods got more attention from the researchers over time. The most important aspect of the feature-based image registration is the extraction of features such as corners, lines, etc. Several feature extraction algorithms have been proposed in computer vision, including SIFT (Scale Invariant Feature Transform) [13], ORB (oriented FAST and rotated BRIEF) [14], BRISK (binary robust invariant salable keypoints) [15] and accelerated (A)-KAZE [16].

**Fig 1:**
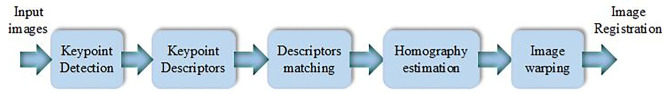
Feature-matching image registration flowchart.

The feature-based image registration process consists of five key processes, shown in Fig 1. The keypoint descriptor matching is the most crucial step in the image registration process. The image registration process adopts additional methods to enhance its performance.

Feature-matching image registration process is classified into two categories based on the selection of image enhancement techniques: intensity-invariant extraction and outlier-removal. Outlier involves the exclusion of outlying features through employing the relation between the features, thus reducing the transformation errors in image registration. LWM (local weighted mean) and RANSAC are used along with the SIFT for outlying feature removal from multimodal image features. However, LWM aided SIFT is not applicble to images with high outlier rates especially optical and SAR images [32]. Another outlier removal model is proposed in [17] where spatial constraint is used to improve the number of inliers that ends in better accuracy of feature matching. However, outlier removal enhancement highly relies on the number of features. If the number of detected keypoints is small, outlier removal technique fails to make improvements in image registration.

Although numerous image registration techniques have been proposed [18–22], However, a key question is still there about the image quality and illumination conditions. In this paper, a novel contrast-enhancement technique is proposed to improve the feature-based image registration process. The key idea is to improve the image contrast [23] using hybrid binary differential evolution and BAT optimization model for low contrast/illumination images. The contrast improvement will thus resulting in enhancing the number of inlier keypoints and features for image registration. In this work, we demonstrate the proposed methodology of contrast enhancement with modeling of hybrid binary differently evolution and BAT optimization. Feature based image registration process using ORB is also explained. The performance of the proposed methodology over low contrast and low illuminated images is shown in the experimental section. Details of the current and future aspects of the proposed research is discussed in the conclusion.

## Proposed methodology

**Fig 2:**
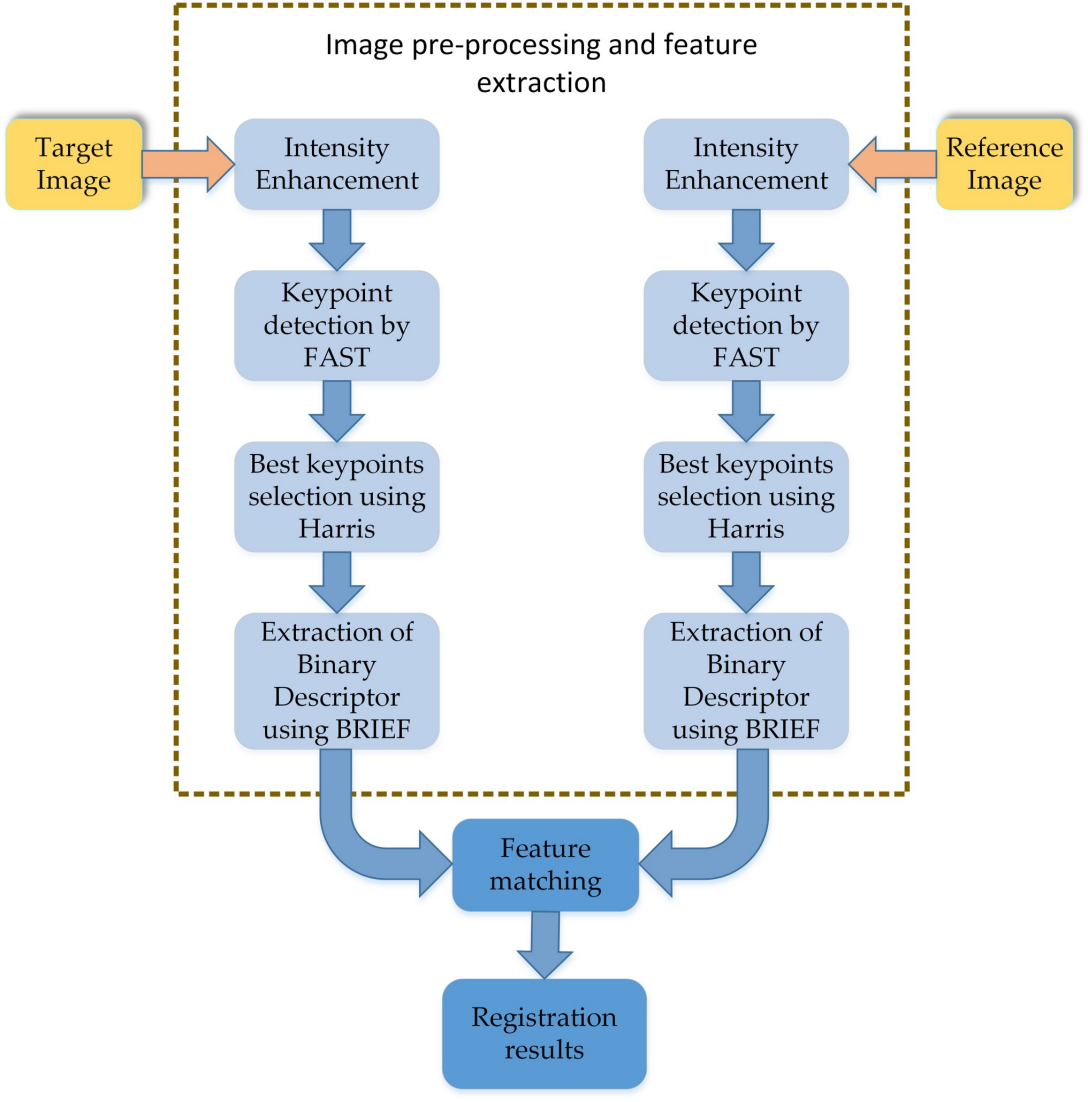
Contrast enhancement aided image registration flowchart.

The proposed methodology for the image registration system, accompanied by contrast stretching, is illustrated in Fig 2. The original target and reference images undergo preprocessing for contrast enhancement with hybrid binary differential evolution and BAT optimization (DeBo). The preprocessed images were subsequently utilized to identify features for image registration.

The introduced contrast stretching DeBo algorithm is comprised of the following steps:

- Convert original RGB images to HSI color space.
- Apply contrast enhancement transformation to intensity pixels only.
- Combine the transformed I(intensity) pixels to original H(hue) and S(saturation) to create HSI image.
- Transform new HSI image to RGB image for further processing.

**Fig 3:**
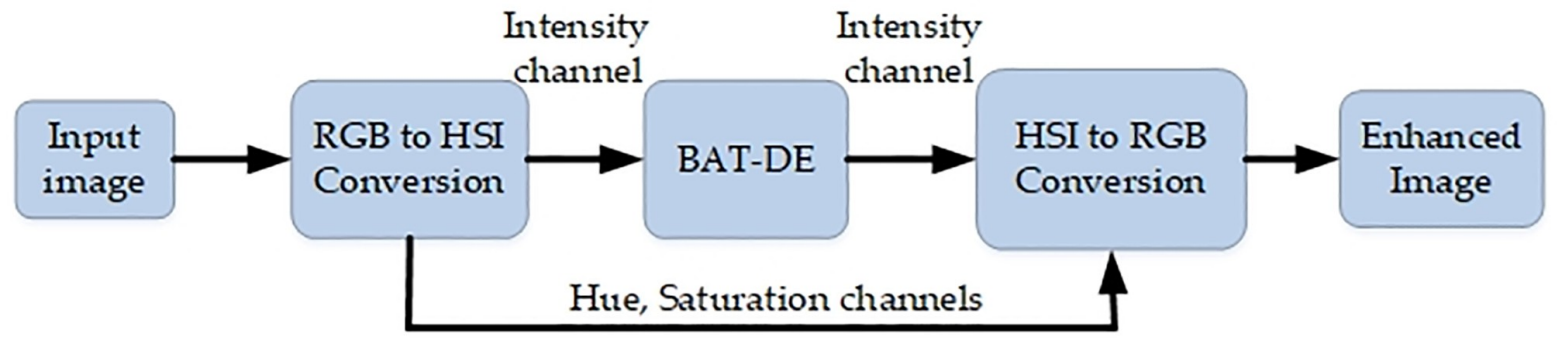
DeBo contrast transformation flow.

The flow of DeBo algorithm for contrast transformation is shown in the Fig 3.

### Contrast transformation cost function

The intensity channel’s contrast is augmented using local area enhancement models, including local mean and standard deviation. The subsequent function represents the contrast enhancement transfer function.

$$\begin{array}{c} \left\{\Psi^{*}\right\}=T_{f}\left(\Psi \right),\;\;\Psi ,\Psi^{*}\in {ℜ}^{M,N} \end{array}$$ (1)

In the aforementioned equation, *T*_*f*_ denotes the transformation function that enhances the intensity of the original image intensity value Ψ, which comprises *M* rows and *N* columns. While traditional techniques like histogram equalization yield superior results, they are computationally intensive. In contrast, we employed a modified statistical method, as outlined in [24], which necessitates fewer computations than the original proposed in [25]. The subsequent function illustrates enhancements in pixel-level intensity.

$$\begin{array}{c} l(i,j)=(\mu \frac{M}{\xi (i,j)+\varepsilon })\times [\kappa (i,j)-\gamma \times \vartheta (i,j)]+\vartheta {\left(i,j\right)}^{\alpha } \end{array}$$ (2)

In the aforementioned equation, *κ*(*i*, *j*) represents the original pixel intensity level, *l*(*i*, *j*) denotes the enhanced version, *M* signifies the global mean, and (*i*, *j*) indicates the central pixel upon which the operation is executed. *ϑ*(*i*, *j*) and *ξ*(*i*, *j*) represent the estimated local mean and standard deviation, respectively, computed from the neighboring pixels of *k* × *k* region. The non-zero value of *ε* enables the utilization of zero standard deviation while employing *γ*, allowing for the selection of a fraction of the mean value for subtraction. The decision variables, *ε* and *γ*, remain constant throughout the processing of an individual image. The contrast enhancement transformation function relies on the automatic estimation of decision variables. This function is employed to modify the intensity of the intensity channel at the pixel level.

$$\begin{array}{c} O_{f}(\Psi^{*})=\frac{\text{log}(\text{log}(E(\Psi^{*})))\times n_{edgels}(\Psi^{*})\times H(\Psi^{*})}{R\times S} \end{array}$$ (3)

here, *R* × *S* represents the dimension of the image. Mainly the objective function is based on the entropy *H*(Ψ*) and intensity *E*(Ψ*) functions of enhanced intensity channel. Sobel edge filter is used for detection of edges and edge intensity [32] using the following equations.

$$\begin{array}{c} E(\Psi^{*})=\sum_{i\in R}\sum_{j\in S}\sqrt{h_{\Psi }{\left(i,j\right)}^{2}+v_{\Psi }{\left(i,j\right)}^{2}} \end{array}$$ (4)

$$\begin{array}{c} \begin{array}{c} h_{\Psi }(i,j)=\upsilon_{\Psi }(i+1,j+1)+2\upsilon_{\Psi }(i+1,j)+\upsilon_{\Psi }(i+1,j-1)-\\ \;\;\;\;\upsilon_{\Psi }(i-1,j-1)-2\upsilon_{\Psi }(i-1,j)-\upsilon_{\Psi }(i-1,j+1) \end{array} \end{array}$$ (5)

$$\begin{array}{c} \begin{array}{c} v_{\Psi }(i,j)=\upsilon_{\Psi }(i+1,j+1)+2\upsilon_{\Psi }(i,j+1)+\upsilon_{\Psi }(i-1,j+1)-\\ \;\;\;\;\upsilon_{\Psi }(i-1,j-1)-2\upsilon_{\Psi }(i,j-1)-\upsilon_{\Psi }(i+1,j-1) \end{array} \end{array}$$ (6)

### Differential evolution

**Fig 4:**
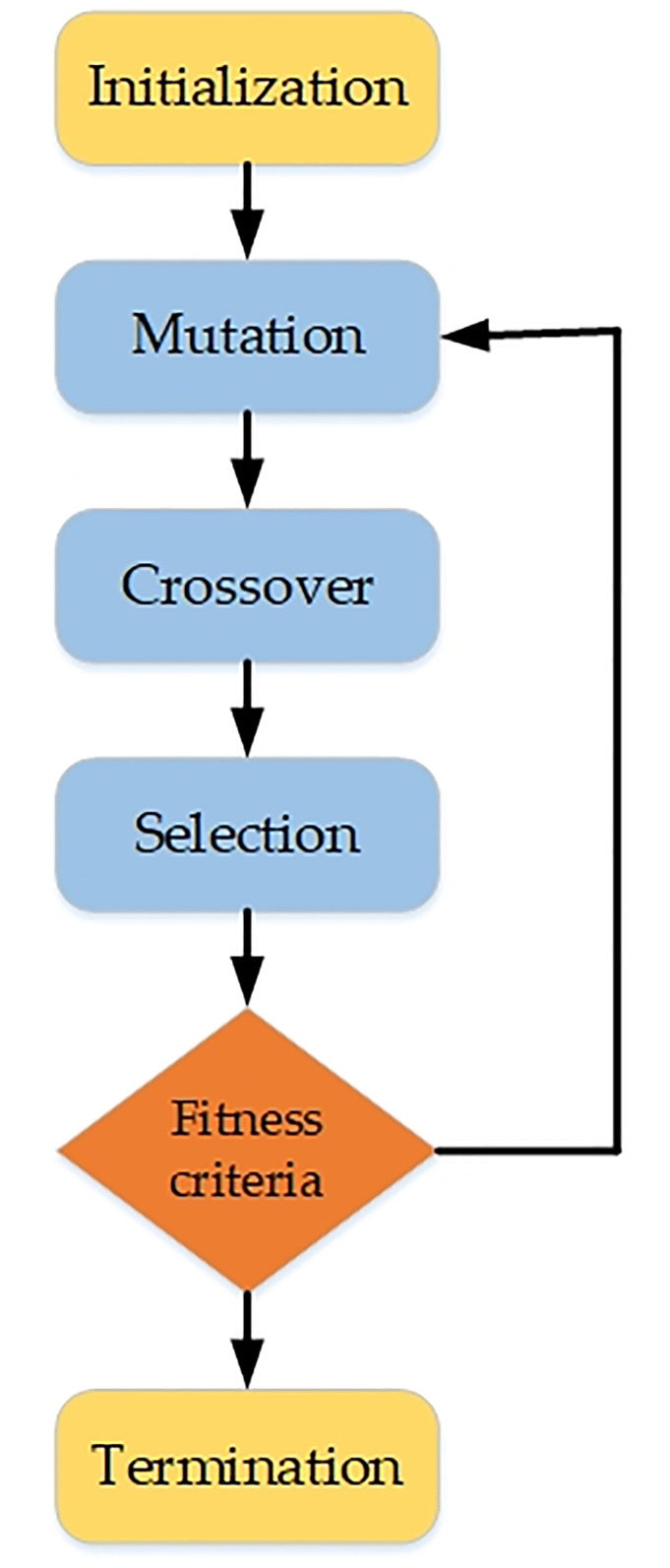
Differential-Evolution process flowchart.

Differential Evolution (DE), introduced in [26], is an evolutionary method that utilizes global search parameters to identify optimal solutions. DE functions in two stages: 1) Initialization; 2) Evolution. During initialization, the population is produced randomly. Throughout the evolutionary process, the created population undergoes mutation, crossover, and selection procedures. The stages are reiterated iteratively to fulfill the selection criteria [27]. The fundamental flowchart of differential evolution is illustrated in Fig 4.

***Initialization***: Initialization involves the generation of uniformly distributed population. Let $\Xi^{G}=\left\{\Phi_{j}^{G}:j=1,2,\ldots ,p\right\}$, where *Ξ*^*G*^ is the search space and $\Phi_{j}^{G}$ is a *D*−dimensional population vector with generation *G* and of size *p* i.e. $\Phi_{j}^{G}=\left\{\phi_{1j}^{G},\phi_{2j}^{G},\ldots ,\phi_{Dj}^{G}\right\}$. Following expression represents the uniform generation of population:

$$\begin{array}{c} \Phi_{j}^{G}=\Phi_{l}+\left(\Phi_{u}-\Phi_{l}\right)*rand\left(0,1\right) \end{array}$$ (7)

where {Φ_*l*_, Φ_*u*_} represents the lower and upper bounds of search space respectively.

***Mutation***: In mutation step, a mutation vector $\Lambda_{j}^{G}$ is generated for every target $\Phi_{j}^{G}$ using the following expression:

$$\begin{array}{c} \Lambda_{j}^{G}=\Phi_{r1}^{G}+\alpha *\left(\Phi_{r2}^{G}-\Phi_{r3}^{G}\right) \end{array}$$ (8)

where *α* ∈ {0, 1} is the scaling factor and {*r*1, *r*2, *r*3} ∈ {1, 2, …, *p*} are randomly selected but mutually different.

***Crossover***: After mutation, a new vector is generated, in crossover step, known as trial vector, $\Psi_{j}^{G}=\left\{\psi_{1j}^{G},\psi_{2j}^{G},\ldots ,\psi_{Dj}^{G}\right\}$. Crossover is performed among mutant vector $\Lambda_{j}^{G}=\left\{\upsilon_{1j}^{G},\upsilon_{2j}^{G},\ldots ,\upsilon_{Dj}^{G}\right\}$ and target vector $\Phi_{j}^{G}=\left\{\phi_{1j}^{G},\phi_{2j}^{G},\ldots ,\phi_{Dj}^{G}\right\}$ using crossover probability *C*_*p*_ ∈ {0, 1}.

$$\begin{array}{c} \psi_{i,j}^{G}=\{\begin{array}{cc} & \upsilon_{i,j}^{G},\text{if}\;rand_{j}<C_{p}\\ & \varphi_{i,j}^{G},\text{otherwise} \end{array} \end{array}$$ (9)

where *i* ∈ {1, 2, …, *D*}.

***Selection***: During the selection process, a comparison is performed between trial vector and target vector with respect to fitness criteria. The operation is performed with the following expression:

$$\begin{array}{c} \Phi_{j}^{G+1}=\{\begin{array}{cc} & \Lambda_{j}^{G},\text{if}\;f\left(\Lambda_{j}^{G}\right)\leq f\left(\Phi_{j}^{G}\right)\\ & \Phi_{j}^{G},\text{otherwise} \end{array} \end{array}$$ (10)

In this paper we adopted the binary differential evolution, proposed in [28].

### BAT optimization

BAT optimization is a metaheuristic algorithm [29]. The term “metaheuristic” combines two Greek words, meaning “to investigate a solution to a higher level”. In other words, metaheuristic techniques are computational models that try to enhance the candidate solutions to a near-optimal level through an iterative process in comparison to a given quality level. The key advantage of meta-heuristics is that they can explore very large spaces of contender solutions by making a few assumptions about the optimization problem. However, the limitation of metaheuristics is their inability to guarantee an optimal solution. Most metaheuristics are nature-inspired and population-based. Following are the key characteristics of the metaheuristic algorithms:

- Metaheuristics are approaches that lead to exploring the search space to extract the near-optimal solution.
- Meta-heuristic-based techniques range broadly from simple local search models to complex learning procedures.
- Meta-heuristic algorithms are non-deterministic approximations.
- Metaheuristics are not problem-specific.

BAT optimization is a metaheuristic algorithm inspired by the hunting behavior of bats. The model is based on the inherent traits of micro-bats, specifically their echolocation and biosonar capabilities. Before discussing the details of the BAT algorithm, first provide some details related to echolocation process of micro-bats.

#### Echo-location process of micro-bats

Approximately 1,000 bat species exist in nature, varying from the diminutive bumblebee bat, which weighs about 2 grams, to larger species that can weigh up to 1 kilogram and possess a wingspan of approximately 2 meters. Echolocation is a natural phenomenon shown by all bats to varying degrees. Nevertheless, among all species, microbats predominantly utilize echolocation.

In micro-bats, echolocation, a type of sonar is used to find preys, detect and avoid obstacles and to find the location of their roosting crevices in the night. Echolocation emits a loud sound like pulse that bounces back from neighboring objects and bats listen these echoes. Different bat species make hunting strategies based on these echo pulses, since they have different properties. Most bats emits short sound pulses, frequency modulated with frequency range of 25kHz-150kHz, and lasts for few milliseconds. Micro-bats emits 10-20 sound waves bursts per second, however, this speed increased up to 200 per second when they are homing to their prey. At a constant frequency, the wavelength, at the velocity *v* = 340*m*/*s* of sound in the air, is ranging from 2mm to 14mm, in the same order of their prey size.

#### BAT inspired optimization model

Xin-She Yang proposed the BAT optimization algorithm in [30] based on the echolocation behavior of micro-bats. The proposed algorithm is the first of its form in terms of natural computational intelligence and optimization problem. In the proposed BA algorithm, every bat is presented using a velocity $v_{i}^{t}$ along with a location $S_{i}^{t}$ for every iteration i and in the g-dimensional search space. The bat location represents the solution of the problem need to be optimized. If the population contains n number of bats, the current near-optimal solution S* is modified every time in iterative process.

The mathematical formulation of original BAT, from [30] by Yang, is represented in the following equations.

$$\begin{array}{c} h_{i}=h_{min}+\left(h_{max}-h_{min}\right)\xi \end{array}$$ (11)

$$\begin{array}{c} v_{i}^{t}=v_{i}^{t}+\left(S_{i}^{t-1}-S^{*}\right)h_{i} \end{array}$$ (12)

$$\begin{array}{c} S_{i}^{t}=S_{i}^{t-1}+v_{i}^{t} \end{array}$$ (13)

Above equations represent the updating process of bat location and velocity in every iteration respectively. *ξ* = [0, 1] is the random vector that is obtained through uniform distribution. Although, in the iterative process, sound pulse rate and loudness can vary, however, the following equations provide a simple relation for the sound wave emission rates and loudness,

$$\begin{array}{c} L_{i}^{t+1}=\kappa L_{i}^{t} \end{array}$$ (14)

$$\begin{array}{c} q_{i}^{t+1}=q_{i}^{0}\left[1-exp\left(-rt\right)\right] \end{array}$$ (15)

where 0 < *k* < 1 and *r* > 0 are constants.

**Algorithm 1** BAT Optimization Algorithm

**Input:** Bat population vector: $S_{i}={S_{i1}∼S_{ik}}^{T}$, for the iterations: i=1,2,…,d

**Output:** The near-optimal solution *S*_*best*_ along *f*_*min*_ = *min*(*f*(*S*))

1: *init*_*bat()*,

2: *eval* = *evaluate*_*the*_*new*_*population*,

3: *f*_*min*_ = *find*_*the*_*best*_*solution*,

4: **while** termination condition not meet **do**

5:  **for** *i* = 1 to *d* **do**

6:   *v* = *generate_new_solution*(*S*_*i*_)

7:   **if** *rand*(0, 1)>*r*_*i*_ **then**

8:    *v* = *improve_the_best_solution*(*S*_*best*_),

9:   **end if** {local search step}

10:   *f*_*new*_ = evaluate_the_new_solution(*v*)

11:   *eval* = *eval*+ 1

12:   **if** *f*_*new*_ ≤ *f*_*i*_ and *N*(0, 1)<*A*_*i*_ **then**

13:    *S*_*i*_ = *v*; *f*_*i*_ = *f*_*new*_

14:   **end if** {save_the_best_solution_conditionally}

15:   *f*_*min*_ = *find*_*the*_*best*_*solution*(*S*_*best*_)

16:  **end for**,

17: **end while**

Algorithm 1 represents the basic BAT optimization pseudocode that is summarized in the following steps:

1. *Initialization* (lines 1:3): The is the first step and involves the initialization of population and algorithm parameters. Moreover, also initial best solution over the given population is also calculated.
2. *New Solution Generation* (line 6): This step involves the movement of virtual bats in the search space based on the BA updating rules.
3. *Local Search* (line 7-9): In this step, best solution is improved based on the Random Walks.
4. *New Solution Evaluation* (line 10): The new solution is evaluated in this step.
5. *The Best Solution Update* (line 12-14): The best solution is archived in this step based on the given conditions.
6. *Discover best Solution* (line 15): The best solution is updated for the current iteration.

## Image registration

As mentioned earlier, feature detection is the first step towards the final image registration. In this work, we used ORB algorithm for feature detection step. ORB algorithm is proposed in [31] by E. Rublee et al. ORB is a combination of two algorithms, FAST and BRIEF where FAST (Features from Accelerated Segment Test) [17] is used for feature detection and modified version of BRIEF (Binary Robust Independent Elementary Features) [32] is adopted as feature descriptor. The ORB based image feature extraction and matching process consists of three steps:

- Extraction of feature points
- Generation of feature point descriptor
- Matching feature points

### Extraction of feature points

Feature points in an image are points that show significant presence, such as bright spots in a dark area or dark spots in bright areas. The ORB algorithm uses FAST for feature point extraction. In FAST, the image pixel is considered the corner point if it is significantly different from its neighborhood pixels. We divide the complete feature point extraction process into the following sub-processes.

**Fig 5:**
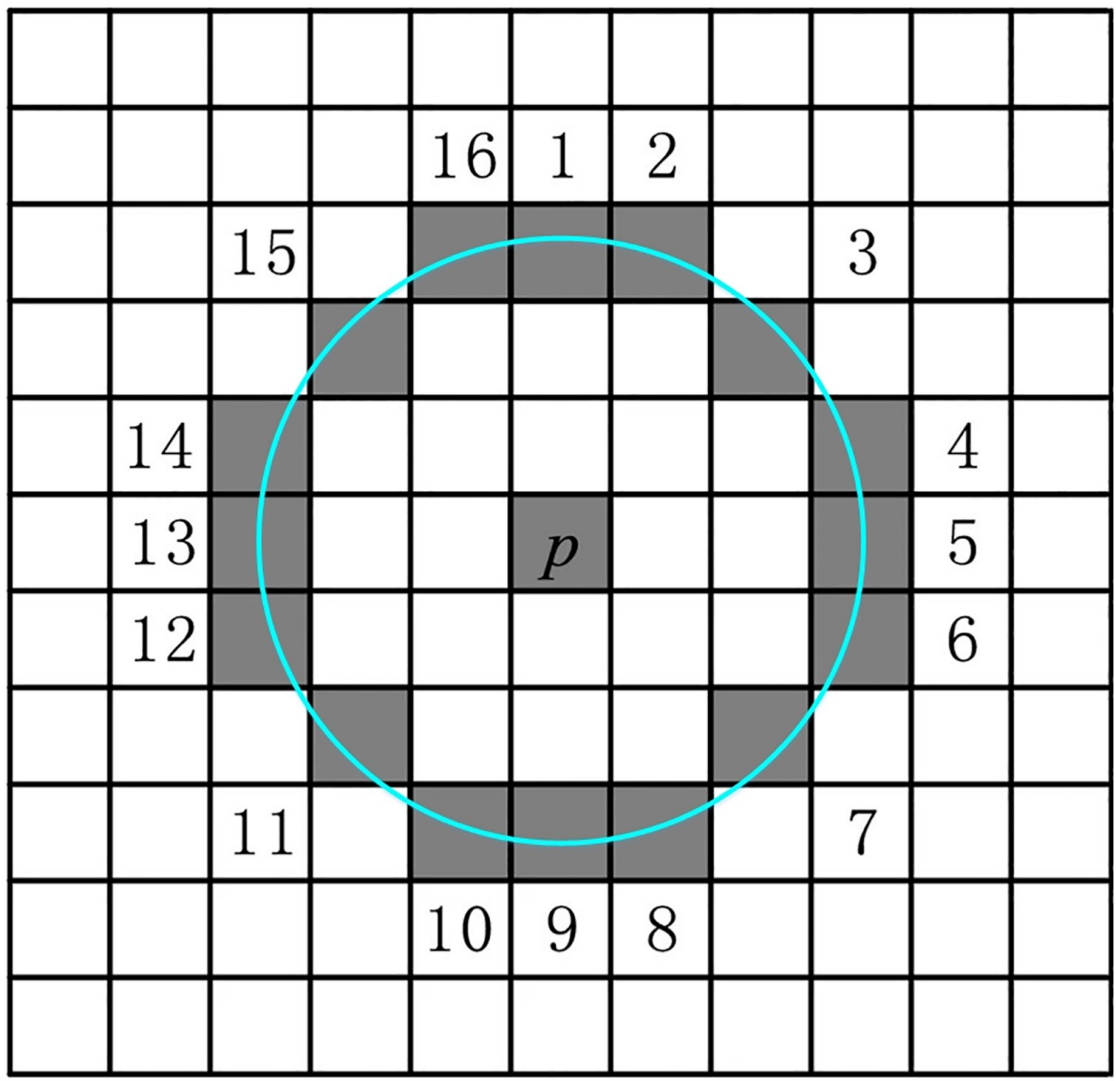
Schematic representation of selection of neighboring pixels in FAST algorithm.

1. At first, a pixel *P* is selected from the image with the brightness of *I*_*p*_. By considering a brightness threshold *T*, the gray value of sixteen neighbor pixels in a circle is compared with the pixel *P* as shown in the Fig 5.
  If the difference of consecutive *N* points on the circle is greater than *ε*_*th*_ or less than *ε*_*tl*_, the pixel *P* is considered as the feature point.
  $$\begin{array}{c} N=\sum_{x\forall (circle(p))}\left(I_{p}-T\right)\varepsilon_{tl}>\left|I_{x}-I_{p}\right|>\varepsilon_{th}\left(I_{p}+T\right) \end{array}$$ (16)
  where *I*_*x*_ is the gray value of the point on the circle. Mostly, three-quarters of the total points on the circle is defined as the *N*. For example if at least 12 points around the circle exceeds the threshold, the center point is considered as corner otherwise it is rejected for feature point.
2. Feature Point Screening: In the original FAST algorithm, a feature point is selected by comparing the brightness value between the pixels; therefore, a large number of feature points are detected with no information related to direction. The modification of the original FAST algorithm in ORB is based on the Harris response value. The following equations calculate the Harris response for all features detected by the original FAST.
  $$\begin{array}{c} R=det\left(M\right)-k{\left(trace\left(M\right)\right)}^{2} \end{array}$$ (17)
  $$\begin{array}{c} M=\sum w\left(x,y\right)[\begin{array}{cc} I_{x}^{2} & I_{x}I_{y}\\ I_{x}I_{y} & I_{y}^{2} \end{array}] \end{array}$$ (18)
  In the above equations, *R* is the Harris response, *M* is a 2x2 matrix, *w*(*x*, *y*) is the image window function, while *k* is a constant ranging between [0.04 0.06]. *I*_*x*_*I*_*y*_ are the feature point variations in the horizontal and vertical directions, respectively. However, the whole process is time consuming when applied to all points. Therefore, we can make a prediction based on the outcomes of four candidate points surrounding the central pixel, separated at 90-degree intervals. We reject a feature point without evaluating its neighboring points unless three out of four points exhibit sufficient gray level difference to verify all the points.
3. Creation of Image Scale Pyramid: The original FAST algorithm, used in the ORB algorithm, does not detect direction related information of feature points. However, the algorithm is modified for rotation invariance and scale invariance through using the grey level intensity centroid technique and by establishing image Gaussian pyramid respectively. At start, the moment of image block i.e. the orientation of the corner in a small image block can be defined as:
  $$\begin{array}{c} \upsilon_{pq}=\sum_{\left(\alpha ,\beta \in \Gamma \right)}\alpha^{p}\beta^{q}\lambda_{\alpha ,\beta }\;\;p,q=0,1 \end{array}$$ (19)
  where *α*, *β* are the coordinates of the image pixel in the neighborhood of the feature point and λ_*α*,*β*_ represents the grey level intensity of the correspondent pixel. The centroid of image block is calculated by the following relation,
  $$\begin{array}{c} \Lambda =(\begin{array}{c} \frac{\upsilon_{10}}{\upsilon_{00}},\frac{\upsilon_{01}}{\upsilon_{00}} \end{array}) \end{array}$$ (20)
  where *υ*_00_ is the 0^th^ moment and represents the image block mass, while the centroid of the image block is represented by the 1^st^ moment of the image block i.e. (*υ*_10_, *υ*_01_). Finally, the orientation of the corner feature point is calculated with the following equation along with the correction if the feature point is dark with respect to its background.
  $$\begin{array}{c} \vartheta =\{\begin{array}{c} tan^{-}1\left(\upsilon_{01}/\upsilon_{10}\right)\;\text{feature}\;\text{point}\;\text{is}\;\text{bright}\\ tan^{-}1\left(\upsilon_{01}/\upsilon_{10}\right)+180\;\text{feature}\;\text{point}\;\text{is}\;\text{dark} \end{array} \end{array}$$ (21)
  The above relations make the FAST feature points rotation and scale invariance.

### Feature points descriptor

The feature descriptor’s goal is to characterize and distinguish the detected feature points. A feature descriptor is numerical information extracted from the image to distinguish one feature point from the others. In the ORB algorithm, an improved version of BRIEF [32] is used to calculate feature descriptors of the detected points. The BRIEF algorithm’s fundamental principle is to calculate the binary feature vector for all detected feature points using FAST. The BRIEF algorithm designates each feature point with a binary feature vector or feature descriptor, a string of 0 and 1, consisting of 128–512 bits. BRIEF’s core idea is based on the principle that it is possible to express the image neighborhood using a small amount of intensity contrast.

$$\begin{array}{c} \tau \left(\lambda ;x,y\right)=\{\begin{array}{c} 1,\lambda_{x}<\lambda_{y}\\ 0,\lambda_{x}\geq \lambda_{y} \end{array} \end{array}$$ (22)

where λ_*x*_ is the gray level intensity of the pixel at position *x* around the feature point while λ_*y*_ is the gray level intensity at position *y* around the feature point. Moreover, Gaussian filtering is also applied at start to reduce the noise effect. Let’s random select a set of N pixels from a *kxk* neighborhood window with feature point at the center, normally N is taken as 128, 256 or 512. Lastly, an N-dimensional vector consisting of N binary strings is calculated as:

$$\begin{array}{c} f_{N}\left(p\right)=\sum_{1\leq i\leq N}2^{i-1}\tau \left(p;x_{i},y_{i}\right) \end{array}$$ (23)

In the BRIEF algorithm, only single pixel image neighborhood is adopted that is susceptible to noise. In order to solve this problem, a *m*x*m* pixel sub-window is used in the ORB with a neighborhood window of size *k*x*k*. In this case, the selection of sub-window obeys the Gaussian Distribution. The original BRIEF is undirected and is not invariant to rotation. A 2*n* matrix is defined for any n criterion feature set at position (*x*_*i*_, *y*_*i*_),

$$\begin{array}{c} Q=\{\begin{array}{c} x_{1},x_{2},\ldots ,x_{n}\\ y_{1},y_{2},\ldots ,y_{n} \end{array}\} \end{array}$$ (24)

A corrected *Q* matrix is constructed using the direction of neighborhood *ϑ* and the corresponding rotation matrix, Υ_*ϑ*_, *Q*_*ϑ*_ = Υ_*ϑ*_*Q*. And the final Steered BRIEF descriptor is:

$$\begin{array}{c} g_{n}\left(p,\vartheta \right):=f_{N}\left(p\right)|\left(x_{i},y_{i}\right)\in Q_{\vartheta } \end{array}$$ (25)

### Feature matching

Feature point matching entails the assessment of similarity between the feature points of two distinct images. After calculating the feature point descriptors, the subsequent step entails estimating the matching of feature points. Numerous feature matching algorithms exist, including Brute Force and Hamming distance. Hamming distance is utilized in BRIEF to quantify the number of differing characters between two strings of same length. Nonetheless, Brute Force is simpler since it calculates the distance between each feature point of image *I*_*t*_ and every feature descriptor of image *I*_*t*_+ 1, and matching features based on the shortest distance. The FLANN (Fast Library for Approximate Nearest Neighbors) method is utilized alongside ORB in certain visual SLAM models [33].

## Experimental results and analysis

The proposed model’s performance is assessed using various pairs of infrared and optical (RGB) images. The testing images vary in resolution, lighting conditions, incident angles, and other factors. The quantity of inlier features is significant, as is the count of accurate matches. Consequently, the quantity of inlier features is assessed prior to and following the application of contrast enhancement. RMSE (root mean square error) [34] is computed to quantitatively assess the registration performance for the matching keypoints. The following mathematical model is employed to compute the RMSE:

$$\begin{array}{c} RMSE=\sqrt{\frac{1}{n}\sum_{i=1}^{n}{\left(x_{i}^{\tau }-x_{i}^{\rho }\right)}^{2}+{\left(y_{i}^{\tau }-y_{i}^{\rho }\right)}^{2}} \end{array}$$ (26)

In the above equation, $\left(x_{i}^{\tau },y_{i}^{\tau }\right)$ represents the pair of *i*_*th*_ matched feature point from target image and $\left(x_{i}^{\rho },y_{i}^{\rho }\right)$ represents the pair of *i*_*th*_ matched feature point from reference image.

**Fig 6:**
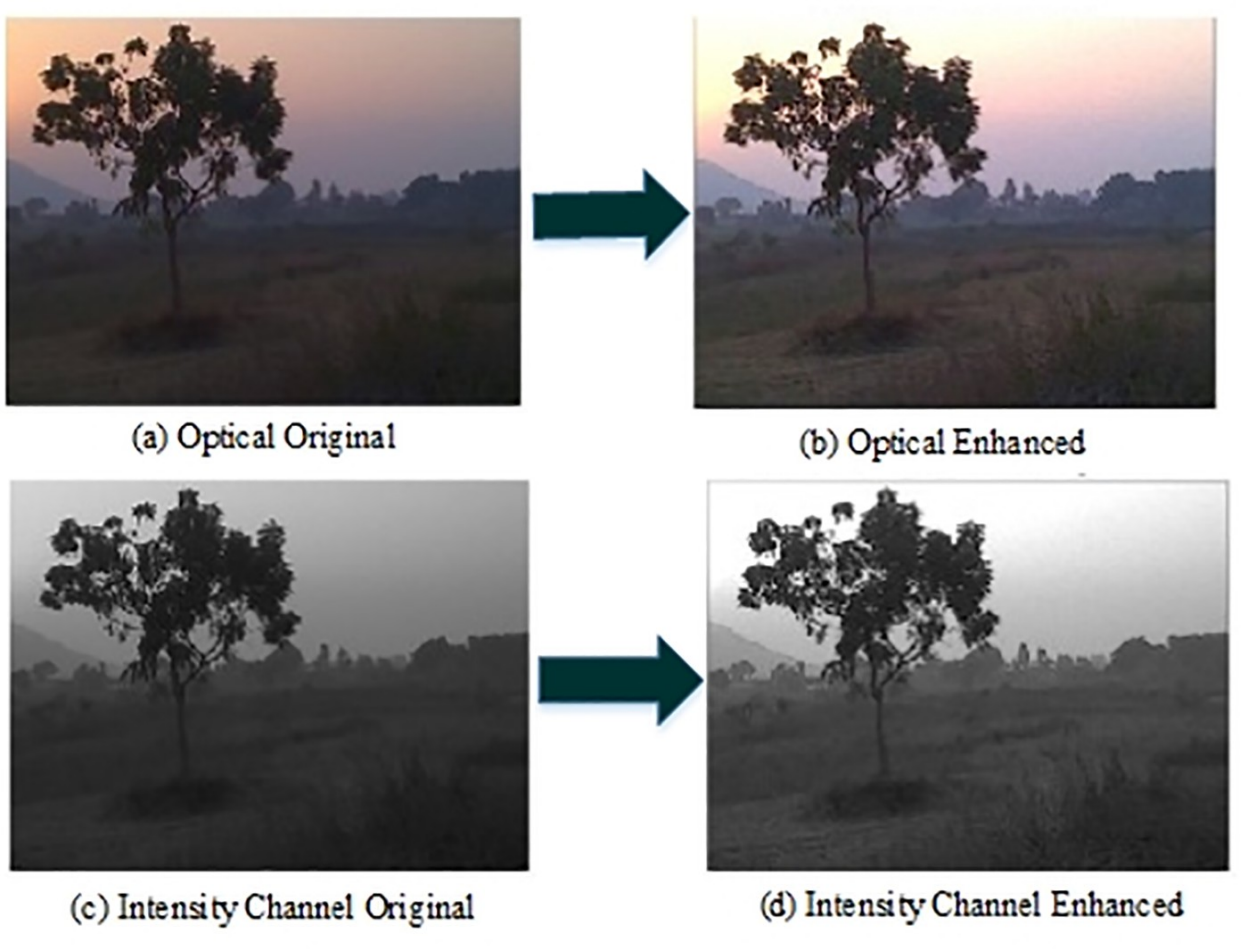
Image contrast enhancement on low light optical images. Figure (a) shows the original optical image under low light condition. Figure (b) represents the contrast enhanced optical image. Figure (c) and (d) represents only original intensity channel of the HSI (hue,saturation,intensity) image and enhanced one respectively.

**Fig 7:**
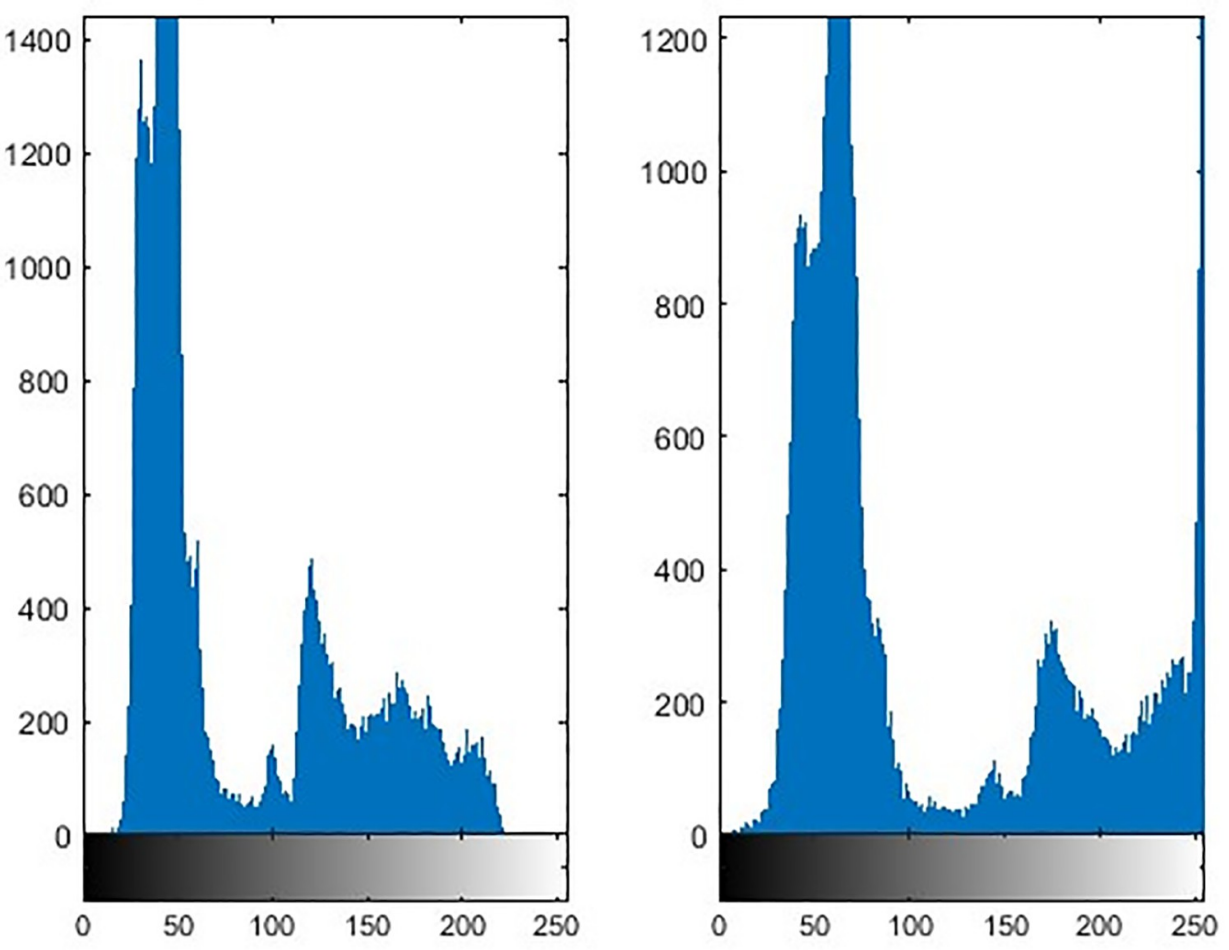
Contrast improvements analysis based on image histogram. Left image represents histogram of optical image before contrast enhancement while right histogram is the representation after contrast enhancement.

**Fig 8:**
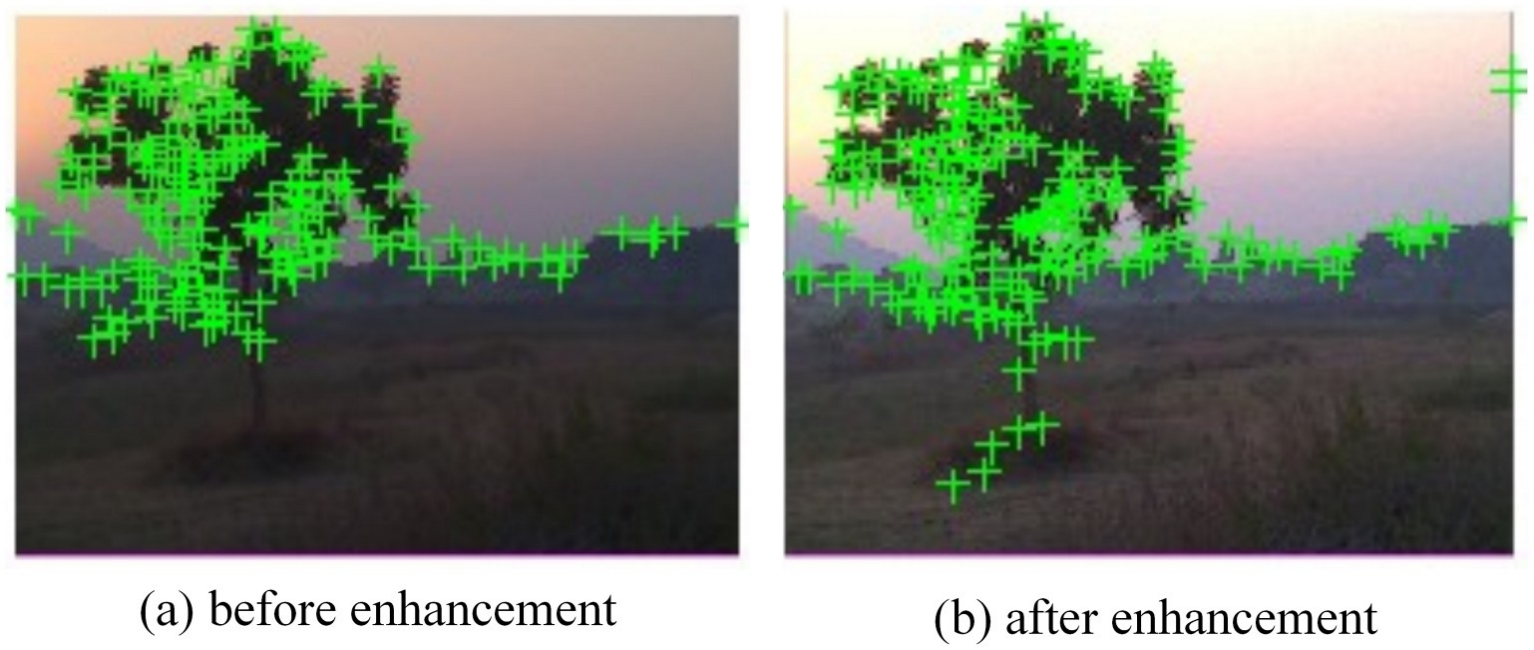
ORB detected features before and after enhancement mapped on the relevant images.

Fig 6 shows the image enhancement results on the intensity channel (d) and optical enhancement (b). The enhancement has significant impact on the number of detected features. The histogram comparison of the Fig 6 is shown in the Fig 7. The left side histogram is prior enhancement. From the figure, it can be analyzed that enhancement model improves the distribution of pixels that can be seen in right side image of Fig 7. In Fig 8 inlier features are shown after RANSAC correction.

Table 1 represents the number of features detected before and after applying the proposed enhancement model in the low light conditions. In this condition number of features are increased for both optical and thermal images before and after applying the RANSAC algorithm. RANSAC algorithm is used for detection and removal of outlier features in image registration process.

**Table 1:** Quantitative analysis of Fig 6.

Image	Method	Features before RANSAC	Registered Features
(a)	ORB	173	50
	Proposed	195	59
(b)	ORB	185	50
	Proposed	210	59

**Fig 9:**
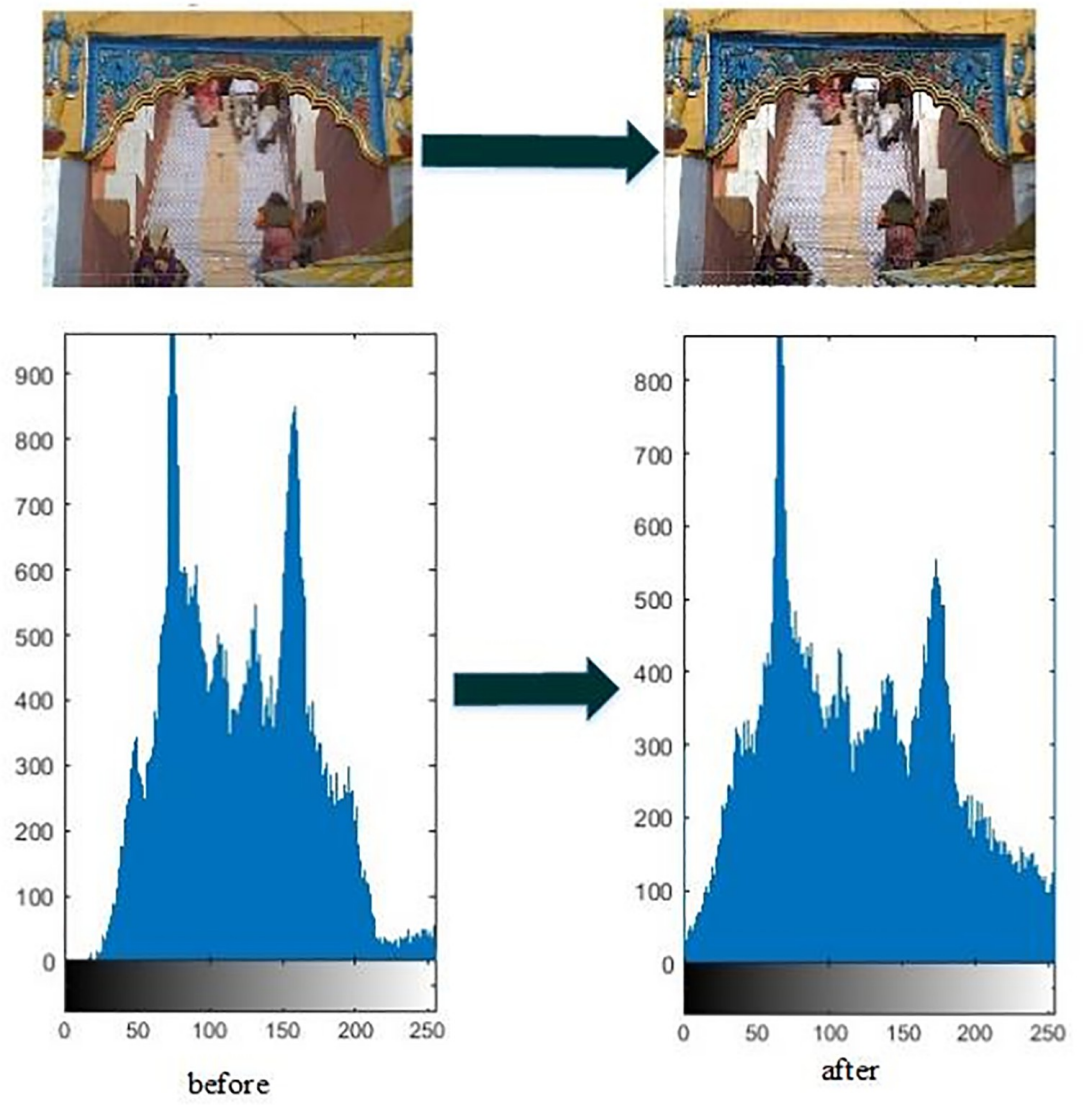
High quality optical image enhancement.

Fig 9 represents the image enhancement results of the high quality optical image. Table 2 represents the corresponding quantitative analysis. Although the number of total detected features are reduced in optical image after applying enhancement, however, there is a significant improvement in the registered number of features (features found in both images).

**Table 2:** RMSE analysis of image registration examples in Fig 9.

Image	Method	registration points	RMSE/pixel
(a)	ORB	30	3.842
	Proposed	38	2.01
(b)	ORB	24	2.746
	Proposed	37	1.8959
(c)	ORB	19	3.9270
	Proposed	31	3.2079

**Fig 10:**
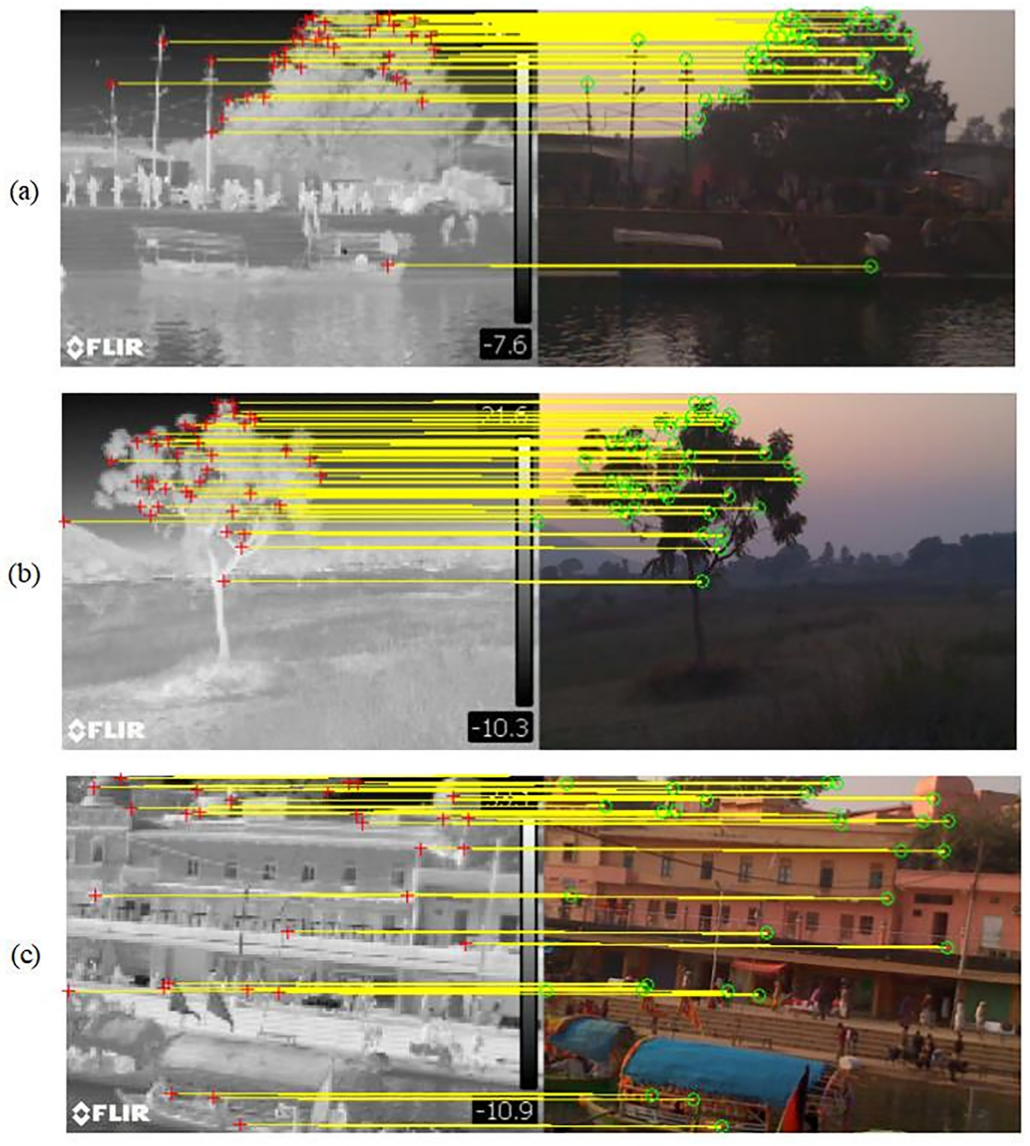
Scene detection through registration. Three optical and thermal image pairs under different environmental conditions. Image pairs (a) and (b) are taken as examples of low light conditions and image (c) is taken in bright light.

Image registration examples are presented in Fig 10 with optical and thermal pair of images. The quantitative analysis of Fig 10 in terms of RMSE is shown in the Table 3. In first two pairs both registration points are significantly increased in addition of significant reduction in RMSE/pixel. However, for third pair, under bright light, RMSE/pixel i not reduced significantly, although, number of registration points are significantly improved.

**Table 3:** RMSE analysis of image registration result shown in Fig 10.

Image	Method	registration points	RMSE/pixel
(a)	ORB	30	3.842
	Proposed	38	2.01
(b)	ORB	24	2.746
	Proposed	37	1.8959
(c)	ORB	19	3.9270
	Proposed	31	3.2079

## Conclusion

In this paper, we introduced an image contrast enhancement step in the image registration process based on a novel hybrid binary differential evolution and BAT optimization model. The results proved the improvements in the image registration process, especially for low-light optical images. The fundamental idea of image contrast enhancement involves enhancing the distribution of pixel constraints through the application of the natural BAT optimization model and differential evolution. The population size plays a crucial role in the optimization model, as the processing time directly correlates with the size of the BAT population. Although an increase in population also increases processing, it has minimal effects on performance results, a topic not covered in this paper. The performance results showed that the proposed model is also effective for bright images. The proposed model helps to increase the number of detection and matching features in the image registration process. Despite the results proving the significance of the model, the performance of the model was significantly affected by the population size during contrast enhancement. A very small population size can result in quick computation at the cost of low improvements in the image quality; conversely, a very high population level can take a long computation time. During this work, we used a fixed population size; in the future, we will develop a model focusing on the dynamic selection of the population size according to image requirements.
